# Analysis of tracheal intubation in out-of-hospital helicopter emergency medicine recorded by video laryngoscopy

**DOI:** 10.1186/s13049-021-00863-9

**Published:** 2021-03-17

**Authors:** Jürgen Knapp, Bettina Eberle, Michael Bernhard, Lorenz Theiler, Urs Pietsch, Roland Albrecht

**Affiliations:** 1 Department of Anaesthesiology and Pain Medicine, Inselspital, Bern University Hospital, University of Bern, Freiburgstrasse, 3010 Bern, Switzerland; 2 Department of Anaesthesiology, Cantonal Hospital of Graubünden, Chur, Switzerland; 3 Emergency Department, Heinrich-Heine-University, University Hospital of Düsseldorf, Düsseldorf, Germany; 4 Department of Anaesthesiology, Cantonal Hospital of Aargau, Aarau, Switzerland; 5 Swiss Air Rescue, Rega, Zurich, Switzerland; 6 Department of Anaesthesiology and Intensive Care Medicine, Cantonal Hospital St. Gallen, St. Gallen, Switzerland

**Keywords:** Videolaryngoscopy, Intubation, Airway, Prehospital emergency medicine, HEMS

## Abstract

**Background:**

Tracheal intubation remains the gold standard of airway management in emergency medicine and maximizing safety, intubation success, and especially first-pass intubation success (FPS) in these situations is imperative.

**Methods:**

We conducted a prospective observational study on all 12 helicopter emergency medical service (HEMS) bases of the Swiss Air Rescue, between February 15, 2018, and February 14, 2019. All 428 patients on whom out-of-hospital advanced airway management was performed by the HEMS crew were included. The C-MAC video laryngoscope was used as the primary device for tracheal intubation. Intubation procedures were recorded by the video laryngoscope and precise time points were recorded to verify the time necessary for each attempt and the overall procedure time until successful intubation. The videos were further analysed for problems and complications during airway management by an independent reviewer. Additionally, a questionnaire about the intubation procedure, basic characteristics of the patient, circumstances, environmental factors, and the provider’s level of experience in airway management was filled out. Main outcome measures were FPS of tracheal intubation, overall success rate, overall intubation time, problems and complications of video laryngoscopy.

**Results:**

FPS rate was 87.6% and overall success rate 98.6%. Success rates, overall time to intubation, and subjective difficulty were not associated to the providers’ expertise in airway management. In patients undergoing CPR FPS was 84.8%, in trauma patients 86.4% and in non-trauma patients 93.3%. FPS in patients with difficult airway characteristics, facial trauma/burns or obesity ranges between 87 and 89%. Performing airway management indoors or inside an ambulance resulted in a significantly higher FPS of 91.1% compared to outdoor locations (*p* < 0.001). Direct solar irradiation on the screen, fogging of the lens, and blood on the camera significantly impaired FPS. Several issues for further improvements in the use of video laryngoscopy in the out-of-hospital setting and for quality control in airway management were identified.

**Conclusion:**

Airway management using the C-MAC video laryngoscope with Macintosh blade in a group of operators with mixed experience showed high FPS and overall rates of intubation success. Video recording emergency intubations may improve education and quality control.

## Background

Safe and effective airway management in out-of-hospital emergency medicine is crucial to the resuscitation and stabilization of critically ill or injured patients. Tracheal intubation remains the gold standard in emergency airway management [1]. However, it is known to be more difficult in the out-of-hospital setting than in an emergency department or operating room [2]. Direct laryngoscopy (DL) is a skill needing more than 150 successful intubations to achieve a reasonable overall success rate of 95% in the in-hospital setting [3, 4]. Data also suggest that an increasing number of intubation attempts is associated with higher rates of adverse events [5–7]. Compared with the in-hospital setting, personnel resources are limited in out-of-hospital emergency medicine (e.g., no back-up by a senior anaesthesist), and paramedics and emergency medical service (EMS) physicians have varying levels of experience in airway management. Therefore, improving safety, intubation success and especially first-pass intubation success (FPS) in these situations is imperative.

Video laryngoscopy (VL) has become widely available in the in-hospital setting, and emerging data seem to indicate that VL might be superior to DL regarding intubation success [8–13]. But even though VL seems to be easier to learn, it is still a complex skill requiring extensive practice to achieve expertise. This is true even when practitioners are trained in DL and when patients are anaesthetized for elective surgery [14]. In addition, there is still disagreement over which type of laryngoscopy should be the first choice pre-hospital, with the decision often being dependent on the operator [15].

In 2018, the Swiss air rescue service Rega introduced video laryngoscopes on all of their helicopters. This provided the unique opportunity to record every intubation procedure and to generate objective and valid data about intubation times, success rates, and difficulties in video laryngoscopic intubations. The primary aim of this prospective observational study was to determine success rates, time needed, and technical difficulties of out-of-hospital tracheal intubations using VL in a “real-world” setting of out-of-hospital emergency medicine, with providers having highly variable expertise in airway management. Our secondary objective was to identify parameters such as operator experience, patient and environmental characteristics, identify specific problems that might influence these variables, and derive practical recommendations for the use of VL in the out-of-hospital setting.

## Methods

This prospective observational cohort study included all tracheal intubations performed by helicopter emergency medical service (HEMS) physicians of the 12 helicopter bases of the Swiss Air Rescue Rega between February 15, 2018, and February 14, 2019.

### Emergency medical service in Switzerland

The structure of the Swiss EMS differs from region to region. In the high alpine regions the rescue helicopter (staffed with one paramedic and one HEMS physician) is usually the only resource available to deliver professional EMS, and is used for an estimated 35% of HEMS missions. In rural areas, frequently an ambulance (staffed with two paramedics) is called to the scene (about 50% of HEMS missions). Either the operation command center alarms a HEMS crew simultaneously (based on the condition reported in the emergency telephone call), or the ambulance crew can call for an HEMS crew to support them. Depending on the ground-based paramedics’ training and qualifications, they are allowed to use either supraglottic airway devices or tracheal tubes for ventilation in the case of cardiac arrest. Usually paramedics are not certified for anesthetic induction. In these cases advanced airway management is only done after arrival of the HEMS crew.

In urban areas a two tiered EMS system is often used. In case of life-threatening injuries or diseases a rapid response vehicle (staffed with one EMS-physician certified for anesthetic induction and advanced airway management and one paramedic) supports the ambulance crew. In these cases a rescue helicopter is called to the scene either primarily by the operations center or secondarily by the ground-based team for the expedited patient transport to trauma or cardiac arrest centers (about 15% of HEMS missions).

### Video laryngoscopes and data collection

The video laryngoscopes (C-MAC, Karl Storz, Tuttlingen, Germany) were equipped with Macintosh blades of size 0 to 4. Hyperangulated blades were not available. The intubation procedure was videotaped, recorded on the integrated memory card of the video screen, and afterwards analyzed by the study authors, who were not involved in the out-of-hospital rescue mission. According to the study protocol, all tracheal intubations by the HEMS crew had to be performed with the video laryngoscope and recording had to begin before the first attempt at tracheal intubation and stop after successful airway management had been performed. Precise time points were recorded to verify the time necessary for each attempt and the overall procedure time until successful intubation. An intubation attempt was defined as the time between the VL blade passing the lips and entering the mouth, and the blade being retracted and removed from the mouth.

Additionally, a questionnaire about the intubation procedure had to be filled out by the HEMS physician after each mission that required tracheal intubation, asking about his or her prior experience in airway management, basic characteristics of the patient, as well as circumstances and environmental factors of airway management. In addition, the operator was asked to rate the difficulty of the tracheal intubation procedure on a scale from 1 (very easy) to 10 (extremely difficult).

### Outcomes

The primary outcome was the rate of FPS. Secondary outcomes included overall success rate, overall time to successful tracheal intubation, number of intubation attempts, airway management-related complications and subjective level of difficulty. Results are presented according to the Strengthening the Reporting of Observational Studies in Epidemiology (STROBE) Guidelines for observational studies.

### Ethics

The study protocol was approved by the Cantonal Ethics Committee of Bern (Bern, Switzerland, ID number: 2017–02104; Chairperson: Professor Dr. Ch. Seiler) on November 30, 2017, and registered with ClinicalTrials.gov (study identifier NCT03929796). Written informed consent from the patient was waived as only pseudonymised data without follow-up of in-hospital outcome were used.

### Statistical analysis

Data were anonymised and handled by three of the study authors in an electronic database (Excel, Microsoft, Redmond, Washington, USA). Descriptive statistics were used and results were tested for normal distribution. Data are presented as median (interquartile range (IQR) and/or range), and proportions as percentages. FPS between groups was compared using the exact binominal test for single proportions or multinomial test (goodness of fit) with adjustment of *p* values for post hoc comparison. All analyses were performed with Stata, version 16.1 (StataCorp, LLC, College Station, Texas, USA). A *p* value < 0.05 was deemed to be statistically significant.

## Results

During the one-year study period, 1199 tracheally intubated patients received medical treatment by the HEMS crews (787 primary missions, 412 inter-hospital transfers). Of these, 428 patients were intubated by the HEMS crew and 316 of these intubation procedures (73.8%) were video-recorded in full, the results of the questionnaire were available for all 428 patients. The median age of the patients was 58 years (0–100 years), 74.5% were male.

### First-pass success and procedural time interval

Overall, the FPS rate was 87.6% (375/428) and the overall success rate was 98.6% (422/428). Table 1 shows the number of patients, FPS and overall duration of the intubation process associated with specific characteristics, indications for tracheal intubation, locations of patient care and environmental conditions. Tracheal intubation failed in six patients. Five of them were successfully oxygenated and ventilated using a laryngeal mask and one with a laryngeal tube. Median time to successful intubation was 31 s (IQR 23 to 44 s, range 11 to 305 s). The median score for subjective difficulty of tracheal intubation was 3 (IQR 2 to 4.5, range 1 to 10). Visibility of vocal cords according to Cormack and Lehane (C/L), as seen on the screen of the laryngoscope, was grade 1 in 51.2%, grade 2a in 37.1%, grade 2b in 7.0%, grade 3 in 4.4%, and grade 4 in 0.2%.

**Table 1:** First-pass success and overall time to intubation in various subgroups

	***n*** (%)	FPS [%]	overall intubation time, median (range) [s]
Predictors of difficult airway management			
•Difficult airway characteristics^*^	163 (38.1)	87.1	34 (12 to 305)
•Facial trauma/facial burns	63 (14.7)	87.3	32 (13 to 148)
•Obesity^**^	123 (28.7)	88.6	31 (12 to 235)
Location			
•indoors, ambulance car	250 (60.4)	92.8	30 (11 to 165)
•street, woods, public places, alpine environment	142 (34.3)	***81.7***^***‡***^	33 (12 to 305)
•snow, ski slope, glacier	22 (5.3)	72.7	30 (16 to 91)
Patient positioning			
•lying on the ground	237 (55.4)	**84.0**^***†***^	33 (11 to 305)
•elevated (on a stretcher, bed etc.)	182 (42.5)	92.9	29 (12 to 471)
Indication			
•trauma	132 (30.8)	86.4	30 (12 to 149)
•non-trauma	96 (22.4)	93.3	29 (14 to 127)
•cardiopulmonary resuscitation	191 (44.6)	84.8	32 (11 to 305)
Environment			
•Rain/snowfall	10 (2.3)	100	30 (14 to 45)
•Darkness	39 (9.1)	92.3	27 (12 to 65)
•Direct solar irradiation	109 (25.5)	***80.7***^***†***^	30 (12 to 172)
Specific video laryngoscopic problems			
•Fogging	106 (24.8)	***61.3***^***‡***^	39 (12 to 305)
•Blood	65 (15.1)	***81.5***^***†***^	44 (14 to 149)
•Vomit	45 (10.5)	91.1	37 (14 to 137)
•Saliva	76 (17.8)	89.5	33 (12 to 235)

^*^intubation with cervical collar or under manual in-line stabilization, mouth opening < 4 cm

^**^rated subjectively by operator as relevant overweight with the potential to impede the intubation process

† *p* < 0.05, exact binominal test for single proportion

‡ *p* < 0.001, exact binominal test for single proportion

**Fig. 1:**
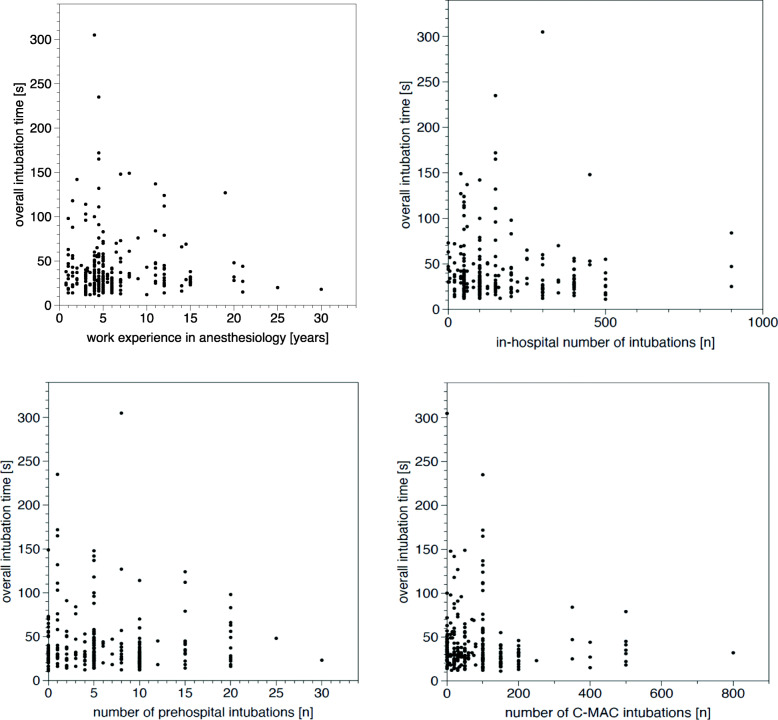
Overall intubation time depending on work experience in anaesthesiology, number of in-hospital intubations during the past year, number of out-of-hospital intubations during the past year and total number of intubations with C-MAC by the corresponding provider

**Fig. 2:**
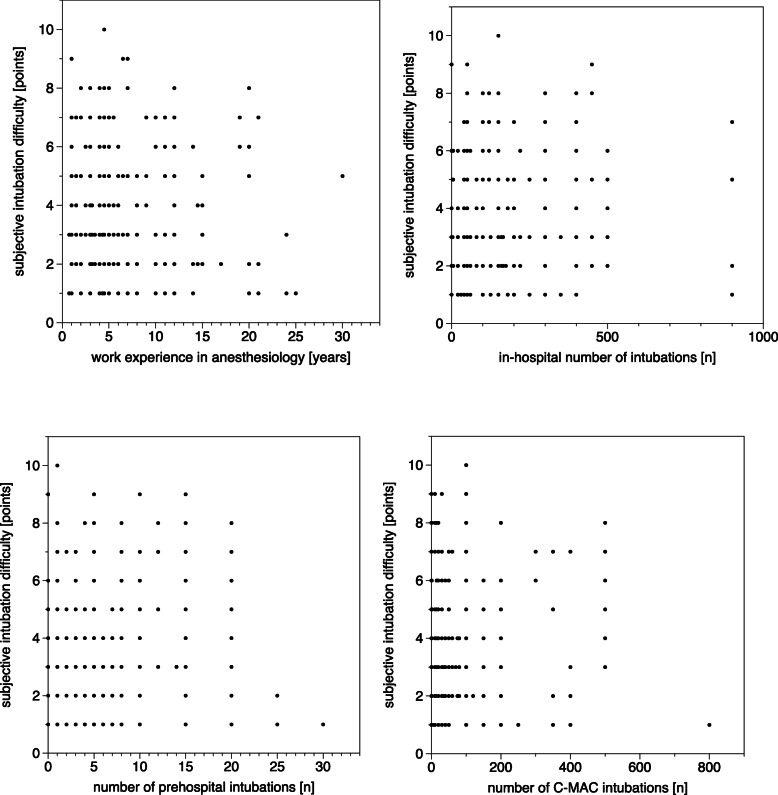
Subjective level of difficulty [score range from 1 (very easy) to 10 (extremely difficult)] depending on work experience in anaesthesiology, number of in-hospital intubations during the past year, number of out-of-hospital intubations during the past year and total number of intubations with C-MAC by the corresponding provider

Airway management was performed by 110 HEMS physicians. Their educational levels and expertise in airway management are listed in Table 2. We found no correlation between the amount of work experience in anaesthesiology or the numbers of previous in-hospital DL-guided or VL-guided intubations in the year prior to the evaluated intubation, and FPS, overall success rate, overall time to intubation or subjective difficulty level of intubation (Table 3**,** Figs. 1 and 2).

**Table 2:** Educational level and expertise in airway management of the intubating HEMS physicians (*n* = 110). Median (range)

Work experience in anaesthesiology [years]	5 (0.75 to 30)
Number of in-hospital intubations during the past year	100 (0 to 900)
Number of out-of-hospital intubations during the past year	6 (0 to 30)
Total number of intubations with C-MAC	35 (0 to 800)
Number of intubations with C-MAC during the past year	10 (0 to 300)

**Table 3:** Work experience of HEMS physicians and corresponding performance (first-pass success and overall intubation time) in tracheal intubation

	***n*** (%)	number of study intubations	FPS [%]	overall time to intubation, median (range) [s]
Work experience in anaesthesiology < 1 year	8 (7)	9	89.5	38 (23 to 63)
Work experience in anaesthesiology < 2 years	13 (12)	47	91.5	36 (14 to 142)
Work experience in anaesthesiology > 10 years	33 (30)	44	84.8	31 (14 to 137)
< 10 tracheal intubations in the year before the study	8 (7)	14	85.7	44 (30 to 73)
> 100 tracheal intubations in the year before the study	46 (42)	189	84.1	30 (11 to 305)

no statistical difference for FPS and overall intubation time between the groups

*FPS* first pass success

### Cardiopulmonary resuscitation

In 150 patients tracheal intubation was performed during cardiopulmonary resuscitation (CPR). In 56 of these patients (37.3%) chest compressions were interrupted for tracheal intubation. The FPS was significantly lower in the group of patients with interrupted chest compressions (67.3 vs. 92.6%, *p* < 0.001), and time to successful intubation was 35 s (range 14 to 305 s) vs. 31 s (range 11 to 124 s) (*p* = 0.02), respectively.

### Video laryngoscopy after unsuccessful intubation attempts by ground-based crews

During the study period the HEMS crews attended 359 further patients in whom a tracheal tube was inserted by ground-based ambulance teams. Three Hundred Thirty-Eight of these tubes were placed correctly in the trachea. Twenty one tubes were placed in the esophagus (5.8%). All of these misplaced tubes were recognized by the intubating teams, and no further intubation attempts were made, as required by the according to the protocols. Subsequently, in 17 of these patients (81.0%) intubation of the trachea by the HEMS crew using VL succeeded on the first attempt. In three patients two further attempts were needed by the HEMS crew and in one patient three further VL-guided attempts were necessary until successful intubation.

### Specific problems of video laryngoscopy

Fogging of the camera lens was observed in 106 cases (24.8%), and led to a significantly decreased FPS (*p* < 0.001, Table 1). If there was direct solar irradiation on the screen, FPS decreased to 80.7% (*p* = 0.02). Saliva or gastric contents on the lens did not alter FPS. In contrast, blood on the lens was associated with a decreased FPS of 81.5% (*p* = 0.02).

After a failed first attempt with an impeded view through the camera, the operator changed from VL to DL (still using the C-MAC laryngoscope) in a total of 10 patients. In eight of these cases the view through the camera was impaired by a combination of secretions (blood, vomit or saliva) on the lens and simultaneously fogging or bright ambient light. In the other two cases the operator reported massive amounts of liquids in the airways in connection with drowning and pulmonary edema, respectively. All patients were intubated successfully on the (in total) second attempt.

In six cases operators reported technical problems with the video function of the laryngoscope (“screen failure” or inability to start the video function). All situations were successfully managed by switching to DL with the C-MAC laryngoscope with Macintosh blade or by restarting the video function of the laryngoscope. Another problem described by one operator was that the insertion of the blade was made more difficult because the camera at the handle of the videolaryngoscope collided with the mechanical chest compression device during the intubation of a patient undergoing CPR.

In 10 cases, a C/L grade 3 or 4 was identified on the screen. Six of these patients’ tracheas were successfully intubated within up to three attempts using additional measures such as lifting the epiglottis with either the tracheal tube or the laryngoscopy blade (“Miller style”), optimizing patient position, using external laryngeal manipulation or using a bougie. In four patients a laryngeal mask was inserted successfully.

### Esophageal intubations using videolaryngoscopy and airway management-related complications

The retrospective analysis of three cases of failed tracheal intubation showed that the tube was positioned in the esophagus. In these three cases, esophageal intubation was only recognized after negative findings on capnometry and/or auscultation. Conversely, the video sequence of an attempted intubation during resuscitation of a neonate clearly showed the tube correctly positioned in the trachea. However, as no capnometric tracing could be detected by the HEMS crew on-site, the tube was removed again and ventilation was attempted by facemask only.

Damage to the mucous membrane caused by the laryngoscope or the tube was observed in 5.4% of the patients. Vomit in the pharynx was seen in the videos of 21.5% overall and in 32.4% of patients undergoing CPR. Ongoing aspiration during airway management or aspirate within the glottis or upper trachea was documented in the videos of 18.0% of emergency intubations.

## Discussion

### Video laryngoscopy in the prehospital setting

To our knowledge, this prospective observational out-of-hospital cohort study of VL-guided tracheal intubation is the first one that provides precise, unbiased objective data for intubation times in the out-of-hospital setting and additionally analyses the intubation procedure. We measured an FPS of 88% and an overall intubation success rate of 99%. Our findings are in line with those of Hossfeld et al. [16], who reported an FPS of 89% and an overall success rate of 100% in trauma patients who were intubated by highly experienced HEMS crews. This is remarkable, as the experience levels in the present investigation involving a total of 110 HEMS physicians were markedly lower on average (Table 2).

The FPS was higher and the overall success rate was comparable to those in a study by Gellerfors et al. [17]. In that study two thirds of the operators were very experienced, with more than 2500 out-of-hospital tracheal intubations performed. A systematic review investigating intubation success in the out-of-hospital setting showed an FPS rate of 79% using DL [18]. Our findings highlight the advantages of VL, especially for relatively inexperienced operators. Furthermore, FPS and median time until successful intubation using VL were comparable in the group of relatively inexperienced (< 1 year working experience in anaesthesiology) and very experienced operators (defined as > 10 years working experience in anaesthesiology) (Table 3, *p* = 0.71). These findings were in line with the results for overall time to intubation by very experienced physicians (consultants in anaesthesiology) in the emergency department (31 ± 9 s) [19]. This also seems to indicate a safety benefit of VL for the out-of-hospital setting.

However, these results should not encourage a reduction in the minimum requirements for qualification in out-of-hospital emergency medicine. In our opinion, the DL technique has to be perfectly mastered, because correct technique is essential for difficult intubations (e.g., small mouth opening, large tongue, neonates, airway swelling), even with the use of VL. The least experienced provider of airway management in our study was a resident with only nine months of experience in anesthesiology. But even that resident had cumulative in-hospital experience of 125 successful tracheal intubations using DL. This minimum number seems to be essential for FPS above 85% in DL under ideal in-hospital conditions [20].

Our “real-world” study with objectively documented intubation conditions revealed several difficulties with the out-of-hospital use of VL. Fogging of the lens was reported frequently. This might be due to our high alpine environment and the corresponding climatic conditions. When fogging was present, the FPS rate was massively impaired (61%). For this reason, in cold ambient conditions we would recommend the use of antifogging agents on the lens to allow prewarming. When blood on the camera lens impaired the view, FPS was reduced to 82% (*p* = 0.02) and median time to intubation was prolonged, whereas saliva and vomit on the lens did not seem to significantly deteriorate intubation conditions compared to conditions in the non-contaminated airway. These results underline that the DL technique still needs to be mastered, as visual problems caused by the camera cannot be excluded. Here, the use of Macintosh blades provides the unique advantage of allowing DL and VL to be used interchangeably. Therefore, our results do not allow the conclusion that VL compensates for a lack of experience in tracheal intubation, and thus minimal requirements for the training of out-of-hospital personnel in airway management should not be lowered, following the recommendations in several national and international guidelines [3, 4, 21].

Prospective, randomized trials on the benefits of VL in the prehospital setting with a heterogenous group of prehospital care providers are still lacking. Prehospital studies on videolaryngoscopic intubation with hyperangulated blades have shown worse success rates compared to DL [22, 23]. This might be explained by the fact that tracheal intubation with a hyperangulated blade is a different technique. Therefore, differences in performance with VL and DL often are operator dependent. In contrast, in our study even providers with little experience in VL-guided intubation performed well, and found airway management as easy as very experienced VL-users. This ease of use could be another benefit of video laryngoscopes with Macintosh blades in prehospital settings where providers have very different levels of training and expertise in airway management: conventional technique is facilitated especially in difficult situations of DL without the need for learning the “hyperangulated technique” [24].

### Video laryngoscopy in cardiac arrest patients

Comparable to the results of our investigation, in prior studies the median time to complete ETI with VL was reported to be between 37 s and 42 s, whereas median intubation times using DL in these studies were 51 s to 62 s [25, 26]. Thisunderlines the benefits of VL, especially in CPR, where short intubation times are critical.

Our results for FPS in patients undergoing CPR exactly match the results of a current study, showing an FPS of 84% in CPR patients intubated by very experienced providers using a video laryngoscope [27]. The difference to FPS in non-CPR patients (90%) was not significant in our study (*p* = 0.14), whereas Hossfeld et al. revealed a significant lower FPS compared to non-CPR patients (in their study 91%, *p* = 0.01). This was probably due to the larger patient population of 1006 in Hossfeld’s study [27]. The reason for the lower FPS in patients undergoing cardiac arrest might be that these patients often have to be intubated in more inconvenient positions: e.g., 83% of our CPR patients were intubated lying on the ground, whereas this was the case in only 34% of the patients not under CPR. And as shown in our study patient positioning on the ground seems to be associated with a worse FPS. These results contradict the old doctrine that tracheal intubation is easier in CPR patients than in other emergency patients. As many EMS license their paramedics for tracheal intubation in cardiac arrest patients our results underline the use of VL also (and perhaps even particularly) for paramedical personnel who typically are less experienced in laryngoscopy than EMS physicians. Additionally, the frequently observed vomit in the pharynx of CPR patients underlines the need for advanced airway management and aspiration protection in these patients.

Our study showed a lower FPS in the subgroups of patients in whom chest compressions were paused for intubation. This may be explained by the fact that chest compressions had to be interrupted more often in patients with difficult conditions for tracheal intubation and conversely, patients with an easy airway were intubated during ongoing chest compressions.

### Video laryngoscopy and quality improvement

The FPS in our study was relatively good compared to international studies. However, it might be further improved by the routine use of a bougie for tracheal intubation. This was documented by Angerman et al. [11], who reported an FPS of 98% when a bougie was used together with a Macintosh blade VL, compared to 86% with VL only.

The use of VL might be associated with a decrease in esophageal intubations [6]. However, as seen in three of our patients when the blade is advanced too deeply into the mouth, the upper esophageal sphincter may be stretched into a longitudinal shape and confused with the glottis, especially by inexperienced users under stress [28]. Such mistakes might be reduced by a dual visual check of tube position (by paramedic and physician), enabled by VL. We cannot evaluate whether this was done in the cases of esophageal intubations reported in our study. The routine recording of tracheal intubations using VL enabled us to demonstrate that such rarely seen complications occur, thereby improving teaching. Therefore, we suggest the use of videotaped intubation procedures for quality improvement in airway management.

In 26% of all cases, the recording function was not activated by the operator. Presumably, due to the stressful situation, the recording button was not pressed, was not held down long enough, or in some cases was pressed twice, so that recording immediately stopped again. Since the end of the study, the manufacturer (Karl Storz, Germany) has introduced an automatic recording function, which we recommend installing. Likewise, the technical problems reported as “screen failure” could be due to the automatic “power off” function in situations where the camera was started several minutes before the intubation procedure (to warm up the lens and avoid fogging). Therefore, we would recommend that the manufacturers either deactivate this function or prolong the latency of “auto power off” in combination with the use of VL.

### Strengths and limitations

To our knowledge, this is the largest and first study analyzing video-recorded intubation procedures in a physician-staffed HEMS system [29, 30]. The strengths of our study are the out-of-hospital and real-life setting and the mixed expertise in airway management of the operators, reflecting the actual situation in large parts of European prehospital emergency medicine. Intubation success and time to intubation were assessed objectively by independent researchers evaluating the recorded videos. The use of the videolaryngoscope was mandatory for all intubations by the HEMS crews of Swiss Air Ambulance during the study period. Due to the central registry of all medical operation records, we were able to identify all patients who were intubated by the HEMS crews. However, in 26% of the cases the video recording was not started correctly or was stopped too early due to operating errors, so that the complete airway management process was not recorded. These cases could not be included in the analysis of time needed for intubation, and data on number of intubation attempts had to be taken from the questionnaire.

## Conclusion

Tracheal intubation using the C-MAC video laryngoscope with Macintosh blade showed high FPS and overall rates of intubation success and seems to be beneficial in a group of providers with very variable expertise in airway management. The possibility to change to DL with the same device is desirable with regard to special conditions in prehospital emergency medicine. Analyzing videorecorded emergency intubation may be beneficial for education and further quality improvement.
